# Isolation and Identification of *Pasteurella multocida* and *Mannheimia haemolytica* from Pneumonic Small Ruminants and Their Antibiotic Susceptibility in Haramaya District, Eastern Ethiopia

**DOI:** 10.1155/2024/5605552

**Published:** 2024-04-16

**Authors:** Mohammed Abdulkadir, Taju Nigussie, Isayas Asefa Kebede

**Affiliations:** ^1^School of Veterinary Medicine, Wolaita Sodo University, P.O. Box 138, Wolaita Sodo, Ethiopia; ^2^School of Veterinary Medicine, Ambo University, P.O. Box 19, Guder, Ethiopia

## Abstract

**Background:**

*Pasteurella* species are frequently encountered as serious diseases in small ruminants. It is the main cause of respiratory pasteurellosis in sheep and goats of all age groups.

**Methods:**

The cross-sectional study was conducted from December 2022 to April 2023 in Haramaya district, eastern Ethiopia, to isolate and identify *Pasteurella multocida* and *Mannheimia haemolytica* and estimate their prevalence, associated risk factors, and antimicrobial sensitivity of isolates in small ruminants using a purposive sampling method. A total of 384 samples (156 nasal swabs from clinic cases and 228 lung swabs from abattoir cases) were collected. STATA 14 software was used to analyze the data. In addition, multivariable logistic regression analysis was performed to assess an association of risk factors.

**Results:**

Out of the 384 samples examined, 164 were positive for pasteurellosis, resulting in a 42.70% prevalence. Similarly, 63 (38.4%) of the 164 positive results were from nasal swabs, while 101 (61.6%) came from lung samples. *M. haemolytica* accounted for 126 (76.82%) of the isolates, while *P. multocida* accounted for 38 (23.17%). Of the 63 nasal swab isolates, 33 (37%) were from goats and 30 (42.8%) were from sheep. And 17 (10.89%) and 46 (29.58%), respectively, were *P. multocida* and *M. haemolytica*. Of the 46 (40%) of the 101 (44.3%) isolates of the pneumonic lung, samples were from goats, while 55 (48.47%) were from sheep. In this study, the risk factors (species, age, and body condition score) were found to be significant (*p* < 0.05). *Pasteurella* isolates evaluated for antibiotic susceptibility were highly resistant to oxacillin (90.90%), followed by gentamycin (72.72%), and penicillin (63.63%). However, the isolates were highly sensitive to chloramphenicol (90.90%), followed by tetracycline (63.63%), and ampicillin (54.54%).

**Conclusion:**

This study showed that *M. haemolytica* and *P. multocida* are the common causes of mannheimiosis and pasteurellosis in small ruminants, respectively, and isolates were resistant to commonly used antibiotics in the study area. Thus, an integrated vaccination strategy, antimicrobial resistance monitoring, and avoidance of stress-inducing factors are recommended.

## 1. Introduction

Ethiopia has a diversified range of animal resources, and its relatively sizable livestock population is well adapted to and spread across different ecological conditions and management methods. It had the most livestock in Africa in 2020, with 65 million cattle, 40 million sheep, 51 million goats, 8 million camels, and 49 million poultry [1]. Small ruminants play a vital role in the nutritional security of millions of rural people, particularly landless smallholder farmers [2].

Small ruminant use and contribution to the national economy are restricted in Ethiopia due to a mix of health issues, inadequate management systems, and starvation [3]. These issues contribute to low reproductive performance in sheep and goats [3–5]. Among the several diseases that afflict sheep and goats, pneumonia is the principal disease restricting the expansion of animal production in the tropics [6]. It is identified as a severe issue typically found in flocks, affecting groups or individuals of all ages and species of sheep and goats [2].

In Ethiopia, pneumonic pasteurellosis is a prevalent respiratory disease that causes epidemics of acute pneumonia in sheep and goats of all ages [3]. This disease reduces sheep production and is one of the most serious infectious diseases of sheep and goats [7–9]. *Pasteurella* species cause the disease, which is a common infection in small ruminants [2]. *M. haemolytica* and *P. multocida* are the most common pathogens of mannheimiosis and pasteurellosis, respectively, and they were identified more frequently from pneumonic animals than from animals without pneumonia [10].

*M. haemolytica* and *P. multocida* are the two most dangerous *Pasteurella* species in the livestock sector. These species live in the animal body as part of the normal nasopharyngeal microflora and are all susceptible to producing infection when the body's defensive mechanisms are compromised [11, 12]. *P. haemolytica* is the most often isolated bacteria from shipping fever, which affects sheep and goats of all ages all over the world [13]. When an animal is stressed by a range of stressors, such as changing weather, shipping (movement), starvation, bacterial invasion of host defense, viral infections, and dehydration, the disease' shipping fever' develops [11, 12].

Despite Ethiopia's small ruminant population's wide range and enormous size, production per animal unit and contribution to the national economy are comparatively low. This might be attributed to a variety of factors, the most significant of which is the increased production inefficiency caused by health limitations such as diseases and treatment resistance. Sheep and goat pasteurellosis is one of Ethiopia's most economically significant infectious diseases [14–18]. Information on the disease's prevalence and related causes, as well as the pathogen involved, is critical in developing disease preventive and control methods. However, there is a paucity of data on the status of the disease in the study area. Thus, the purpose of this study was to estimate the prevalence, isolate, and identify *M. haemolytica* and *P. multocida* associated with pneumonic small ruminants exhibiting respiratory symptoms and their antibiotic profiles in the Haromaya town, eastern Hararghe zone, Oromia, Ethiopia.

## 2. Materials and Methods

### 2.1. Study Area

**Figure 1:**
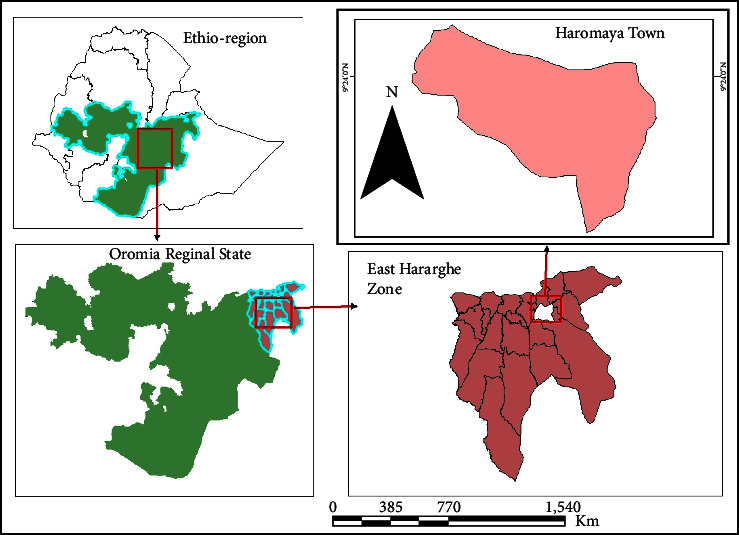
Map of the study area (ArcGIS Software, 2024).

The research was carried out in Haramaya district, in the eastern Hararghe zone of the Oromia regional state, Ethiopia (Figure 1). Haromaya town is 14 km west of Harar and 508 km east of Addis Ababa, the capital city of Ethiopia. The elevation in the region ranges from 1600 to 2100 m above sea level. The District's rainfall is bimodal, with short rain from February to May and lengthy rain from June to September, followed by a dry season from October to February. The average annual rainfall is 492 mm, with ranges ranging from 118 to 866 mm. The average maximum and minimum temperatures are 17 and 9, respectively. Small ruminants are widely grown in large production systems. According to the Haramaya district office of Agriculture and Rural Development [19], there are 193,334 animal populations, including 64,510 cattle, 28,359 goats, 18,930 sheep, 15,277 donkeys, 5 mules, 530 camels, and 65,723 poultry.

### 2.2. Study Animals

The research animals were pneumonic sheep and goats from the Haromaya Veterinary Clinic and healthy sheep and goats slaughtered in the Haromaya municipal abattoir. Sheep and goats are often grown in this area as part of a large production system and are housed with other animal species. All goats and sheep of all ages and sexes with respiratory distress who presented to the study areas of the local veterinary clinic and Haromaya municipal abattoir were included.

Sheep and goats exhibiting respiratory distress such as uneven breathing patterns, grunting on expiration, coughing, dyspnea, inappetence, lethargy, and serous to mucopurulent nasal discharges with fever were believed to be pneumonic [20]. The lungs of the slaughtered animals were observed, palpated, and extensively examined at the abattoir before the pneumonic lungs were evaluated. The animals having pneumonic lungs at the abattoir and the aforementioned clinical symptoms at the veterinary clinic were deemed the study animals in both cases [4]. The age of animals was categorized based on the owner's information and dentation; hence, the two age groups were young (<2 years) and adults (≥2 years) [4].

### 2.3. Study Design and Sampling Techniques

From December 2022 to April 2023, a cross-sectional study was conducted to isolate and identify *Pasteurella* species, as well as to estimate the prevalence, antimicrobial susceptibility test, and associated risk factors of sheep and goat pasteurellosis. Purposive sampling was used to obtain nasal and lung swabs, which were then submitted for bacteriological isolation and identification of *Pasteurella* species.

Thus, 384 samples (156 deep nasal swabs and 228 lung swabs) were collected from small ruminants (222 goat and 162 sheep) for the study; nasal swabs from animals showing signs of respiratory distress and lung swabs from slaughtered animals exhibiting characteristic respiratory macroscopic changes.

### 2.4. Sample Collection and Transportation

#### 2.4.1. Clinic Samples (Nasal Swabs)

Each animal was recognized, confined, and fixed by an assistant. After disinfecting the outside of the nose with 70% alcohol, a sterile cotton-tipped swab was placed into the nostril and rotated against the wall of the nasal cavity. The swab was put in a labeled sterile test tube containing 3 ml of tryptose Soya broth and preserved on an icebox for transit to Haramaya University's Veterinary Microbiology Laboratory [21]. Briefly, the clinic samples were collected following observed evidence from the field showing that there has been frequent occurrence of respiratory diseases suggestive of pneumonic pasteurellosis in sheep and goats in the study area. Those sheep and goats brought to each Haromaya town veterinary clinic were examined for the clinical evidence of pneumonia and those that were observed with anorexia, coughing, dyspnea, lethargy, bilateral nasal discharge, and abnormal lung sounds were considered to have pneumonia, and thus sampled.

#### 2.4.2. Abattoir Samples (Lung Swabs)

Following the slaughter of the healthy animal being stunned mechanically, the lungs were all examined using the normal postmortem meat inspection process. During the examination, the surface of each suspicious lung was incised with a sterile scalpel blade, and the inner surface of the incision was sampled with a sterile swab. The swab was submitted to Haramaya University's Veterinary Microbiology Laboratory using the same method as the nasal swab [22].

### 2.5. Bacteriological Swab Sample Examination and Isolation

#### 2.5.1. Cultural characteristics of *Pasteurella* Species

*Pasteurella* was isolated and identified at Haramaya University's Veterinary Microbiology Laboratory using protocols recommended by Hardy Diagnostics, Santa Maria, CA, USA [23]. The isolation and identification process began with incubating the pre-enriched tryptose Soya broth specimen for 24 hours at 37°C. After 24 hours, a loop of broth cultures was removed and streaked over an identifiable Petri plate containing blood agar base enriched with 7% sheep blood, and then incubated aerobically at 37°C for 24 hours [22]. Second, representative colonies from culture-positive plates were stained with gram stain to evaluate staining responses and cellular morphology under a light microscope at 100x magnification. Mixed and Gram-negative bacteria were carefully subcultured on blood (Oxoid, UK) and MacConkey (Oxoid, UK) agar plates for further examination [22].

The growth of typical colonies on both blood (Oxoid, UK) and MacConkey agar (Oxoid, UK) was described using blood agar for the presence of hemolysis, the type of hemolysis, the general appearance of colonies (morphology, color, shape, size, and consistency), and the ability to ferment lactose [20]. The growth cultures of colonies with features of round (smooth), greyish hue, small to moderate size, and mucoid consistency that was either haemolytic or nonhaemolytic on blood agar (Oxoid, UK) and MacConkey agar (Oxoid, UK) were identified. *M. haemolytica* was identified as having narrow beta hemolysis on blood agar and growth on MacConkey agar with lactose fermentation. Those that were nonhaemolytic on blood agar base and did not grow on MacConkey agar were classified as *P. multocida* [4, 22].

#### 2.5.2. Biochemical Tests for the Identification of *Pasteurella* Species

Pure cultures of a single colony type were put onto nutrient agar slants for a variety of basic biochemical assays, including catalase, oxidase, and fermentative/oxidative tests [22, 24]. Secondary biochemical tests for metabolic end products and fermentation of sugars; glucose, lactose, maltose, sucrose, and arabinose were used to identify the bacteria at the species level [22, 25]. If the organism is unable to generate indole, *M. haemolytica* is suspected. If the organism can generate indole, it is thought to be *P. multocida* [20, 24].

### 2.6. Antimicrobial Susceptibility of *Pasteurella*

Kirby Bauer's disk diffusion method was used to determine antibiotic susceptibility. The isolate's pure culture colony suspension was made with sterile physiological saline and adjusted to 0.5 McFarland standards before being spread to Muller Hinton agar (Oxoid, UK) with sterile cotton swap and allowed to stand for 3–5 minutes to observe any excess moisture from the medium before applying the antimicrobial discs. The ring of each disc containing a single concentration of each antimicrobial agent (Oxoid, Basing Stoke, and UK) was then gently pressed with the point of the forceps to ensure complete contact with the agar surface and left for 30 minutes for antibiotic diffusion in the disc. The plates were flipped upside down and incubated at 37°C for 18 to 24 hours. The clear zones formed by antibiotic suppression of bacterial growth were measured in mm using a measuring caliper and interpreted as susceptible, intermediate, and resistant according to Clinical and Laboratory Standards Institute breakpoints [26]. Each isolate was evaluated against routinely used antimicrobials for the treatment of pneumonia. These antibiotics were chloramphenicol (30 *µ*g), penicillin-G (10 IU), ampicillin (10 *μ*g), gentamycin (30 *µ*g), kanamycin (30 *μ*g), oxacillin (30 *µ*g), and tetracycline (10 *μ*g).

### 2.7. Data Management and Analysis

The data gathered and recorded on specially developed forms and prepared for analysis were loaded into a Microsoft Excel spreadsheet. All statistical analyses were carried out with the STATA statistical software version 14 (Stata Corp., 4905 Lakeway Drive, College Station, Texas). The number of *Pasteurella*-positive animals that were examined was used to compute the prevalence. The correlation of pasteurellosis (dependent variable) with various independent variables (age, sex, body condition score (BCS), and sample types) was investigated using univariable logistic regression analysis. Multicollinearity was assessed for predictors with a liberal *p* value (*p* ≤ 0.25), and covariates with Kruskal gama values between −0.6 and + 0.6 were included for multivariable logistic regression analysis. Using log-likelihood and Wald statistics, the final model was created using the stepwise backward exclusion procedure. Hosmer and Lemeshow statistics, as well as the Receiver Operating Curve (ROC), were employed to verify model fit and validity [27]. The degree of precision was set at 95%, while the level of significance was set at *p* < 0.05.

### 2.8. Ethical Considerations

A documented letter with the appropriate consent and authority from the School of Veterinary Medicine, Wolaita Sodo University, was received to perform the study. The researcher notified the Haromaya town government agriculture office of the study's purpose and relevant facts. Animals' welfare was considered properly and sufficient care was taken to minimize discomfort, distress, or pain during sample collection. The samples were carried out by trained and experienced vets.

## 3. Results

### 3.1. Overall Prevalence of Isolates

**Figure 2:**
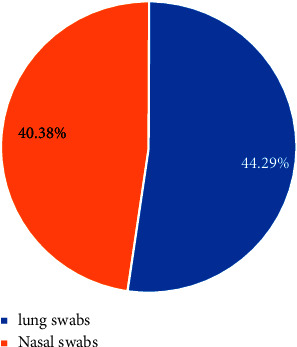
Sample wise prevalence.

The current study revealed that *pasteurella* isolates were more prevalent in lung swabs (44.29%), yielding an overall prevalence of 42.70% (164/384) in the sheep and goats studied (Figure 2).

**Figure 3:**
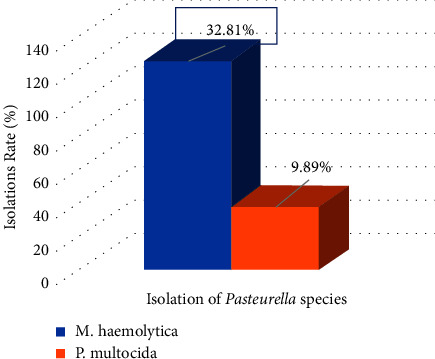
Isolated *Pasteurella* species.

This finding indicates that the proportion of isolated *M. haemolytica* (32.81%) was higher than that of *P. multocida* (Figure 3). Furthermore, 63 (38.4%) and 101 (61.58%) of the 164 *Pasteurella* positives were isolated from sheep and goat nose and lung swabs, respectively. *M. haemolytica* accounted for 76.8% of the isolate, whereas *P. multocida* accounted for 23.17% (Table 1).

### 3.2. Analysis of Pasteurellosis with Associated Risk Factors

Similarly, the greatest prevalence was found in poor body condition (55.55%), followed by adult (50.32%), ovine (48.14%), lung swaps (44.29%), and male (43.67%) when compared to counterparts (Table 2).

The factors studied in the univariable logistic regression analysis of pasteurellosis presence were species, sex, age, BCS, and sample types. Both BCS and age were shown to be significantly (*p* < 0.05) associated with pasteurellosis (Table 3).

The following collinearity testing, all variables in the first analyses with *p* ≤ 0.25 were subjected to a stepwise backward multivariable logistic regression analysis. Accordingly, in the final model, species, age, and BCS variables were revealed to be significant (*p* < 0.05) predictors of pasteurellosis. The Hosmer–Lemeshow goodness-of-fit test suggested that the model fit the data (*χ*^2^ = 39.64; Prob > *χ*^2^ = 0.1979, AUC = 61.64%) (Table 4).

### 3.3. Cultural and Biochemical Characteristics of Isolated *Pasteurella* Species

*P. multocida* isolates were spherical and smooth (mucoid), unable to grow on MacConkey agar, and nonhaemolytic on blood agar, whereas *M. haemolytica* grew as a tiny red colony on MacConkey agar and demonstrated hemolysis on blood agar. *M. haemolytica* and *P. multocida* were both Gram-negative, nonmotile, and coccobacillary on gram staining. To identify the isolated pathogen, several biochemical tests were performed, and the findings showed that both species were positive for catalase, oxidase, and nitrate, and could ferment glucose, mannose, and sucrose. Both *Pasteurella* isolates tested negative for urease. *M. haemolytica* isolates could not produce Indole, but *P. multocida* isolates could produce Indole but could not ferment lactose or maltose.

### 3.4. Antimicrobial Sensitive Test

Table 5 displays the antimicrobial susceptibility data of isolates from pneumonic sheep and goats in this study. A panel of seven antimicrobials was used to test 11 *Pasteurella* isolates from the clinic and abattoir. In this study, *M. haemolytica* and *P. multocida* isolates were found to be highly susceptible to chloramphenicol (80.00%, 100.00%) followed by tetracycline (60.00%, 66.66%) and ampicillin (60.00%, 50.00%), respectively. These isolates, however, were extremely resistant to oxacillin (80%, 100%), gentamycin (80.00%, 66.66%), and penicillin G (60%, 66.66%), respectively.

## 4. Discussion

Pneumonic pasteurellosis is one of the most economically important infectious diseases of ruminants and has a global prevalence. *M. haemolytica* and *P. multocida* are significant bacteria that cause respiratory infections in small ruminants as main or secondary pathogens [28–30].

The total occurrence of small ruminant pasteurellosis in pneumonic sheep and goats was 42.70% in the current study, with 38.7 and 48.14% prevalence in goats and sheep, respectively. *P. multocida* prevalence was nearly the same in the abattoir (9.2%) and clinic (10.89%); however, *M. haemolytica* prevalence was greater at the abattoir than clinic (35% vs 29.49%). This discrepancy of isolated species might be attributed to transportation stress or a great distance from the abattoir, as well as shipment conditions (lung swabs) in the case of the abattoir. That is, animals are transported across long distances, and if the vehicle is overloaded, stress and close contact between animals occur, facilitating the transmission and spread of the agents among animals [31].

In this study, pasteurellosis prevalence (42.70%) was higher than in previous studies by Yeshwas et al. [32], Alemneh and Tewodros [4], Tilaye [33], and Marru et al. [20], which reported rates of 33.1%, 32.6%, 28.4%, and 25%, respectively. However, the current finding was lower than that of Abera et al. [34]; and Hailu et al. [29] who reported 50.2, and 78.38, respectively. This might be attributed to the sample size, the study area, and the period (season) of sampling [28]. Also, this discrepancy might be owing to differences in management strategies and environmental factors.

According to the current findings, *M. haemolytica* was the highest isolated isolate with 76.8%, while *P. multocida* accounted for 23.2% of the total positive results. These findings are congruent with the findings of Alemneh and Tewodros [4], who found an *M. haemolytica* prevalence of 79.5%. Furthermore, this agrees with the findings of Abera et al. [34]; and Ugochukwu [30]. Conversely, unlike an earlier report by Tesfaye [31] on lung infection, the prevalence of *M. haemolytica* isolates in the current study was higher, and it was lower than that of Hailu et al. [35] and Marru et al. [20], who reported 91.03% and 87.5% of *M. haemolytica* as positive results, respectively. Unlike in our study, *M. haemolytica* in goats was not prominent in another study [36]. This difference might be due to *M. haemolytica* being more related to stress and other risk factors. The prevalence and extent of these risk factors vary by location, including husbandry techniques [10].

*M. haemolytica*, a normal flora of the upper respiratory tract, may play a secondary role when the main starting agent inhibits the host defense system and promotes *Pasteurella* species proliferation, resulting in bronchopneumonia in solely pneumonic animals [37]. The causes of stressed animals' higher vulnerability to *M. haemolytica* infection are generally ascribed to stress breakdown of innate pulmonary immunological barriers [38].

According to the current study, 38 (9.89%) of 162 sheep and 222 goat swab samples were positive for *P. multocida. P. multocida* positivity was found in 13 (5.8%) goats and 25 (15.4%) sheep. This finding is inconsistent with previous research, which found *P. multocida* in (16.6%) of lung samples collected from pneumonic sheep and goats in the Fars region of Iran [39]. Similarly, *P. multocida* was a highly isolated organism (31.7%) from infected sheep's pneumonic lungs [25]. Furthermore, Demissie et al. [40] found that the overall isolation rates of *M. haemolytica* and *P. multocida* from pneumonic sheep and goats were 28% and 2.2%, respectively. This discrepancy might be attributed to various methods of collecting samples from merely pneumonic animals, improved healthcare, laboratory facilities, and environmental factors [30].

In comparison to goats (38.7%), sheep (48.14%) had a considerably greater isolation percentage of pasteurellosis (*p* < 0.05, OR = 1.548; 95% CI: 1.013–2.367). This is in line with the findings of Alemneh and Tewodros [4]. However, the results of this study were lower than those reported by Rasha et al. [41], who reported *pasteurella* species from sheep and goats with a recovery rate of 56% and 44%, respectively. Also, this finding was higher than the finding obtained by Alemneh and Tewodros [4], who reported 37.1% and 21.9% prevalence in sheep and goats, respectively.

This disparity might be attributed to differences in sampling methodology and the collection of samples from apparently healthy and solely pneumonic sheep and goats. Similarly, the variation in the prevalence of the two species might be attributed to differences in ruminant grazing behavior. Sheep, which are mostly deep grazers, pick up more germs and hence have a higher exposure than goats, which primarily ingest browse [42, 43].

The current study also found that young age groups (50.3%) of sheep and goats had a greater risk of infection than adults (37.66%) (*p* < 0.05) and there is an association between age and sheep and goat pasteurellosis. This finding is consistent with the findings of Marru et al. [20] and Alemneh and Tewodros [4], which reveal a substantial relationship between age and the prevalence of pasteurellosis in small ruminants. Moreover, pneumonic pasteurellosis occurs in sheep and goats of all ages, with lambs and kids being the most vulnerable during their first life, and dams at lambing. This might be because the immunological condition of animals can predispose them to bacterial infections and other etiological factors [34].

The in vitro disc sensitivity test revealed that chloramphenicol is the most effective antibiotic in the research area, followed by tetracycline and ampicillin. In support of our findings, Marru et al. [20] and Muktar et al. [44] reported chloramphenicol and tetracycline to be the drugs of choice in the treatment of pasteurellosis. *M. haemolytica* and *P. multocida,* on the other hand, showed resistance to oxacillin, gentamycin, and penicillin G. The resistance to penicillin revealed in this study is consistent with the studies of Marru et al. [20], El-Seedy et al. [45], and Girma et al. [46], demonstrating the widespread use of this antibiotic in veterinary practices. Penicillin is an essential antibiotic that has long been used in livestock [3, 47].

The widespread use of these drugs and their improper administration may have led to the development of resistance by *Pasteurella* isolates against this antibiotic in the area. Furthermore, the current findings were similar to the earlier research by Khalili [48], which stated that chloramphenicol is a very effective drug while tetracycline had modest activity against the tested isolates. The current study, however, contrasted with the findings of El-Seedy et al. [45] and Guo et al. [49], who showed that Tetracycline was resistant to *pasteurella* isolates; this might be owing to increasing administration of this antibiotic for the treatment of respiratory disorders in animals.

### 4.1. Limitation of the Study

Because this study was conducted over a short period, we did not observe seasonal fluctuations in the isolates. Furthermore, due to a lack of capabilities in our laboratory, the molecular characterization of the serotypes was not carried out in this work. Aside from these limitations, our study has the following strengths: the sample was taken from both the abattoir and the clinic, demonstrating the trustworthiness of the data; all laboratory work followed standard procedures; and quality control was demonstrated at every stage of the work.

## 5. Conclusions

The results of this study revealed *M. haemolytica* and *P. multocida* were highly prevalent in the study area. *M. haemolytica* is the most common cause of ovine and caprine pasteurellosis than *P. multocida.* The current study found that animal species and age were substantially linked with disease prevalence in sheep and goats. Furthermore, the disease was more common in sheep than in goats, and it was more common in young animals than in adults. This study found that the *Pasteurella* species identified were resistant to routinely used antibiotics. Thus, in the study area, enhancing management methods, vaccination and/or treatment programs, and reducing stress factors should be encouraged. The precise role of the bacterial species, as well as the common serotypes/strain identifications, should be studied further. National regulations and guidelines on the reasonable use of antibiotics are required.

## Figures and Tables

**Table 1:** *Pasteurella* species-wise prevalence.

Samples types (sampling sites)	*M. haemolytica*	*P. multocida*	Total
Lung swabs (abattoirs)	80	21	101 (61.58%)
Nasal swabs (clinics)	46	17	63 (38.41%)
Total	126 (76.82%)	38 (23.17%)	164

**Table 2:** Prevalence of isolated *Pasteurella* species in the study area (*n* = 384).

Variables	Categories	No. of examined	No. of positives	%	95% CI
Species	Ovine	162	78	48.14	40.51–55.87
	Caprine	222	86	38.73	32.52–45.34

Sex	Male	226	95	43.67	36.10–51.54
	Female	158	69	42.03	35.73-48.60

Age	Young	153	77	50.32	42.41–58.22
	Adult	231	87	37.66	31.61–44.11

BCS	Poor	27	15	55.55	36.52–73.08
	Medium	192	90	46.87	39.88-53.98
	Good	165	59	35.75	36.52–73.08

Sample types	Lung swabs	228	101	44.29	37.94-50.84
	Nasal swabs	156	63	40.38	32.93-48.31

CI = confidence interval; % = prevalence.

**Table 3:** Univariable logistic regression analysis of the association of pasteurellosis with different factors.

Variables	Categories	No. of positives	%	OR	95% CI for OR	*p* value
Species	Ovine	78	48.14	1.468	0.974–2.212	0.066
	Caprine	86	38.73	Ref	—	—

Sex	Male	95	43.67	1.069	0.709–1.611	0.750
	Female	69	42.03	Ref	—	—

Age	Young	77	50.32	1.676	1.108–2.536	0.014
	Adult	87	37.66	Ref	—	—

BCS	Poor	15	55.55	2.245	0.985–5.115	0.054
	Good	59	35.75	1.585	1.035–2.427	0.034
	Medium	90	46.87	Ref	—	—

Sample types	Lung swaps	228	101	1.173	0.776–1.774	0.447
	Nasal swaps	156	63	Ref	—	—

OR = odds ratio; Ref = referent category; CI = confidence interval; % = prevalence.

**Table 4:** Multivariable logistic regression analysis of the association of pasteurellosis with potential risk factors.

Variables	Categories	No. of positives	%	OR	95% CI for OR	*p* value
Species	Ovine	78	48.14	1.548	1.013–2.367	0.043
	Caprine	86	38.73	Ref	—	—

Age	Young	77	50.32	1.603	1.050–2.446	0.029
	Adult	87	37.66	Ref	—	—

BCS	Poor	15	55.55	2.969	1.208–7.293	0.018
	Good	59	35.75	1.591	1.028–2.462	0.037
	Medium	90	46.87	Ref	—	—

OR = odds ratio; Ref = reference category; CI = confidence interval; % = prevalence.

**Table 5:** Antimicrobial susceptibility pattern of bacteria isolated from nasal swabs and pneumonic lungs in goat and sheep.

Antimicrobial	Performance	Species of bacteria tested		
		*M. haemolytica* (*n* = 5)	*P. multocida* (*n* = 6)	Total (*n* = 11)
Ampicillin (10 *µ*g)	Susceptible	3 (60.00%)	3 (50.00%)	6 (54.54%)
	Intermediate	2 (40.00%)	2 (33.33%)	4 (36.36%)
	Resistant	0 (0.00%)	1 (16.66%)	1 (9.09%)

Chloramphenicol (30 *µ*g)	Susceptible	4 (80.00%)	6 (100.00%)	10 (90.90%)
	Intermediate	1 (20.00%)	0 (0.00%)	1 (9.09%)
	Resistant	0 (0.00%)	0 (0.00%)	0 (0.00%)

Kanamycin (30 *µ*g)	Susceptible	1 (20.00%)	4 (66.66%)	5 (45.45%)
	Intermediate	4 (80.00%)	2 (33.33%)	6 (54.54%)
	Resistant	0 (0.00%)	0 (0.00%)	0 (0.00%)

Pencilling G (10 IU)	Susceptible	2 (40.00%)	2 (33.33%)	4 (36.36%)
	Intermediate	0 (0.00%)	0 (0.00%)	0 (0.00%)
	Resistant	3 (60.00%)	4 (66.66%)	7 (63.63%)

Tetracycline (10 *µ*g)	Susceptible	3 (60.00%)	4 (66.66%)	7 (63.63%)
	Intermediate	1 (20.00%)	1 (16.66%)	2 (18.18%)
	Resistant	1 (20.00%)	1 (16.66%)	2 (18.18%)

Gentamycin (30 *µ*g)	Susceptible	0 (0.00%)	0 (0.00%)	0 (0.00%)
	Intermediate	1 (20.00%)	2 (33.33%)	3 (27.27%)
	Resistant	4 (80.00%)	4 (66.66%)	8 (72.72%)

Oxacillin (30 *µ*g)	Susceptible	0 (0.00%)	0 (0.00%)	0 (0.00%)
	Intermediate	1 (20.00%)	0 (0.00%)	1 (9.09%)
	Resistant	4 (80.00%)	6 (100.00%)	10 (90.90%)

## Data Availability

All the datasets generated or analyzed during this study are included in this manuscript.

## Abbreviations

CLSI: Clinical Laboratory and Standards Institute
CSA: Central Statistical Agency
HADB: Haramaya Woreda Agricultural Development Bureau.
